# A model of healthy aging based on smartphone interactions reveals advanced behavioral age in neurological disease

**DOI:** 10.1016/j.isci.2022.104792

**Published:** 2022-08-05

**Authors:** Enea Ceolini, Iris Brunner, Johanna Bunschoten, Marian H.J.M. Majoie, Roland D. Thijs, Arko Ghosh

**Affiliations:** 1 Cognitive Psychology Unit, Institute of Psychology, Leiden University, Wassenaarseweg 52, Leiden 2333, the Netherlands; 2 IRIS Brunner, Hammel Neurocenter and University Research Clinic, Aarhus University, Aarhus, Denmark; 3 Department of Neurology, Leiden University Medical Centre, Leiden, the Netherlands; 4 Stichting Epilepsie Instellingen Nederland (SEIN), Heemstede, the Netherlands; 5 Department of Neurology, Academic Centre for Epileptology, Epilepsy Centre Kempenhaeghe & Maastricht University Medical Centre, Maastricht, the Netherlands; 6 MHeNS, School for Mental Health and Neuroscience, Department of Psychiatry and Neuropsychology, Maastricht University Medical Centre, Maastricht, the Netherlands; 7 School of Health Professions Education, Faculty of Health, Medicine and Life Sciences, Maastricht University, Maastricht, the Netherlands; 8 UCL Queen Square Institute of Neurology, London, UK

**Keywords:** Computing methodology, Health technology, Neuroscience

## Abstract

Smartphones offer unique opportunities to trace the convoluted behavioral patterns accompanying healthy aging. Here we captured smartphone touchscreen interactions from a healthy population (N = 684, ∼309 million interactions) spanning 16 to 86 years of age and trained a decision tree regression model to estimate chronological age based on the interactions. The interactions were clustered according to their next interval dynamics to quantify diverse smartphone behaviors. The regression model well-estimated the chronological age in health (mean absolute error = 6 years, R^2^ = 0.8). We next deployed this model on a population of stroke survivors (N = 41) to find larger prediction errors such that the estimated age was advanced by 6 years. A similar pattern was observed in people with epilepsy (N = 51), with prediction errors advanced by 10 years. The smartphone behavioral model trained in health can be used to study altered aging in neurological diseases.

## Highlights

• A smartphone data-driven model was trained to estimate chronological age
• The model trained in health performed well to estimate the age
• The same model estimated advanced aging in stroke and epilepsy
• Smartphone-based model of healthy behavior may help understand aging in diseases

## Graphical abstract

**Figure:**
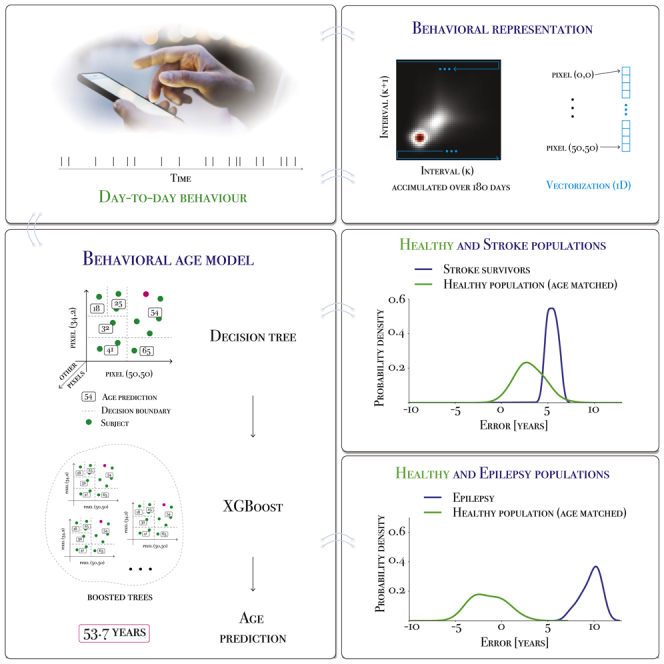

## Introduction

The impact of aging on biology and behavior is complex. Interestingly, quantifying the impacts reveals substantial similarities in how people age. Some of these similarities have been leveraged to build a range of models that can estimate chronological age by using biological measurements, from blood samples to structural scans of the brain (Baecker et al., 2021; Belsky et al., 2020; Fedintsev et al., 2017; Marquand et al., 2016; Schultz et al., 2020). The age-estimating (normative) models contribute toward making the otherwise complex biological measurements interpretable and in parallel provide an opportunity to detect disease-induced deviations from healthy aging (Marquand et al., 2016). For instance, chronological age can be estimated by machine learning models based on rich structural MRI scans obtained from individuals sampled across the life span (Cole et al., 2017). The gap between the estimated age and the chronological age increases in diseases such as stroke and epilepsy as if the diseased brains are “older” than healthy brains (Baecker et al., 2021). According to subjective self-reports, real-world behavior also undergoes complex alterations with age, but these largely unstructured measurements are difficult to leverage toward quantitative modeling of aging in health and diseases (Ding et al., 2018; Lawton, 1971; Testa and Simonson, 1996).

Real-world data stemming from the ubiquitous use of smartphones can be used to quantify and model the behavioral impact of healthy aging. The time series of smartphone touchscreen interactions (tappigraphy) contains information on sleep, diurnal rhythms, the brain’s reward pathways, sensorimotor process, and abnormal brain events in epilepsy (Balerna and Ghosh, 2018; Borger et al., 2019; Duckrow et al., 2021; Huber and Ghosh, 2021; Westbrook et al., 2021). Interestingly, the speed of touchscreen keyboard interactions declines with age and can be used to infer chronological age (Vesel et al., 2020; Zulueta et al., 2021). By organizing the time series of smartphone interactions (across all apps) according to their next interval dynamics, we recently discovered that in healthy adults age-related behavioral alterations vary according to the underlying temporal dynamics (Ceolini et al., 2022). The probability of short (∼100 ms) consecutive intervals declines with age, whereas that of the long (∼4s) intervals increases with age. These observations revealed strong correlations with chronological age and help pave the way toward generating a normative model of healthy aging based on smartphone behavioral inputs.

In this study, we recorded the timestamps of smartphone touchscreen interactions by using a background app (on the subject’s device) in a sample of healthy individuals spanning the adult life span. The timestamp at the onset of any touchscreen interaction event on the screen—may it be a swipe or a tap—across all apps was recorded. The interactions were accumulated for a minimum of 7 days and up to 180 days. These data were subsequently clustered according to the next-interval dynamics resulting in a joint interval distribution (JID). The JID used here captured the diverse behaviors in 2,500 two-dimensional bins and provided an interpretable separation of behaviors—for instance, consecutive short intervals (say due to typing) are represented in distinct two-dimensional bins as opposed to interactions that involve transitioning from a long interval (say due to reading) to a short interval (say due to typing) (Ceolini et al., 2022; Duckrow et al., 2021). Next, we used decision tree regressions to generate a healthy aging behavioral model such that chronological age could be estimated based on the smartphone behavioral JID. The explainability of the model was leveraged to identify which forms of smartphone behavior—in terms of the next-interval dynamics—were utilized for the age estimations. We deployed the normative data-driven model on people with epilepsy and stroke survivors to address the impact of these conditions on behavioral aging. Using this approach, we demonstrate the presence of rich age-related information distributed in real-world behavior in health and capture the distinct pace of behavioral aging in neurological disease.

## Results

### Normative smartphone behavioral model of healthy aging

**Figure 1:**
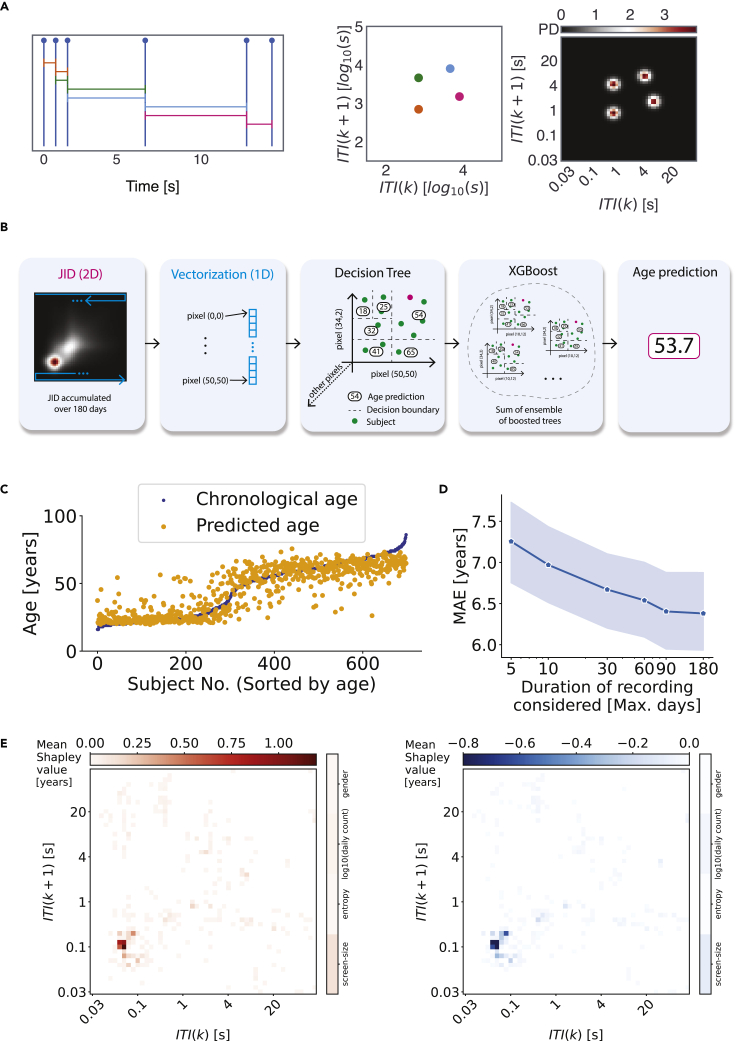
A normative model of healthy aging based on smartphone touchscreen interactions (A) We accumulated the time series of smartphone interactions (tappigraphy) and quantified the next-interval dynamics using a joint interval distribution (*JID*). In this distribution, the inter-touch intervals (*ITI*) are clustered according to the underlying temporal dynamics. (B) XGBoost used the vectorized JID to estimate the chronological age. (C) The model predictions (predicted age) in comparison to the real age (chronological age) are based on unseen data, accumulated over 10 folds of the model training. (D) The impact of the maximum duration of the recordings on the model performance. The mean absolute error (MAE) is shown and the 95% confidence intervals are shaded. (E) The (mean Shapley value across the population) importance of the different features in estimating the chronological age was captured by using the SHAP method.

We gathered smartphone data from self-reported healthy adults. The generic quality-of-life questionnaire, SF-36, was further used to assess the overall health status. The mental component score of the sampled population was 80 (median, 10 interquartile range [iqr]), and the physical component score was 75 (median, 13 iqr, Figure S1). These values were well above what is reported in diseases (Riazi et al., 2003). For the behavioral model to estimate chronological age, the time series of smartphone touchscreen interactions were accumulated for a maximum duration of 180 days. The next-interval dynamics were quantified using a JID and used as model input (Figure 1), along with the gender, the number of interactions per day, the entropy of the JID, and the screen size (Figure S1). According to the predictions generated using 10-fold cross-validations, the XGBoost model performed with a mean error (ME) of −0.53 years and mean absolute error (MAE) of 6.38 years, and the predicted and real chronological ages were strongly related (R^2^ = 0.79). Note that we report the ME (apart from the MAE) as we later compare this measure to the populations with stroke and epilepsy where a ME > 0 would be indicative of accelerated aging.

The explainability of the XGBoost model was leveraged to derive the Shapley values to reveal which of the input features were used by the model and if they contributed to a positive (i.e., to increase the predicted age) or negative (i.e., to reduce the predicted age) output (Figure 1). The two-dimensional bins capturing the rapid consecutive intervals showed both positive and negative Shapley values indicating that the sub-second rapid interactions were prominently used by the model to arrive at the age estimates. Interestingly, slower interactions disproportionately contributed to higher age estimation in the model (positive Shapley values at the slower intervals but only sparsely present negative values at the same intervals). Notably, gender, screen size, overall smartphone usage, or JID entropy played a negligible role in the age estimation in contrast to the JID two-dimensional bins.

We addressed the amount of data necessary for the normative model trained using the maximum of 180 days of data (median 117 days). When the model was provided with less than 90 days of data during the testing phase, the performance was marginally worse (Figure 1). Still, with 5 days of recording, the model performed with a ME of −1.28 years and MAE of 7.25 years, and the predicted and real chronological ages were strongly related (R^2^ = 0.76).

### Age estimation using the normative model in stroke survivors

**Figure 2:**
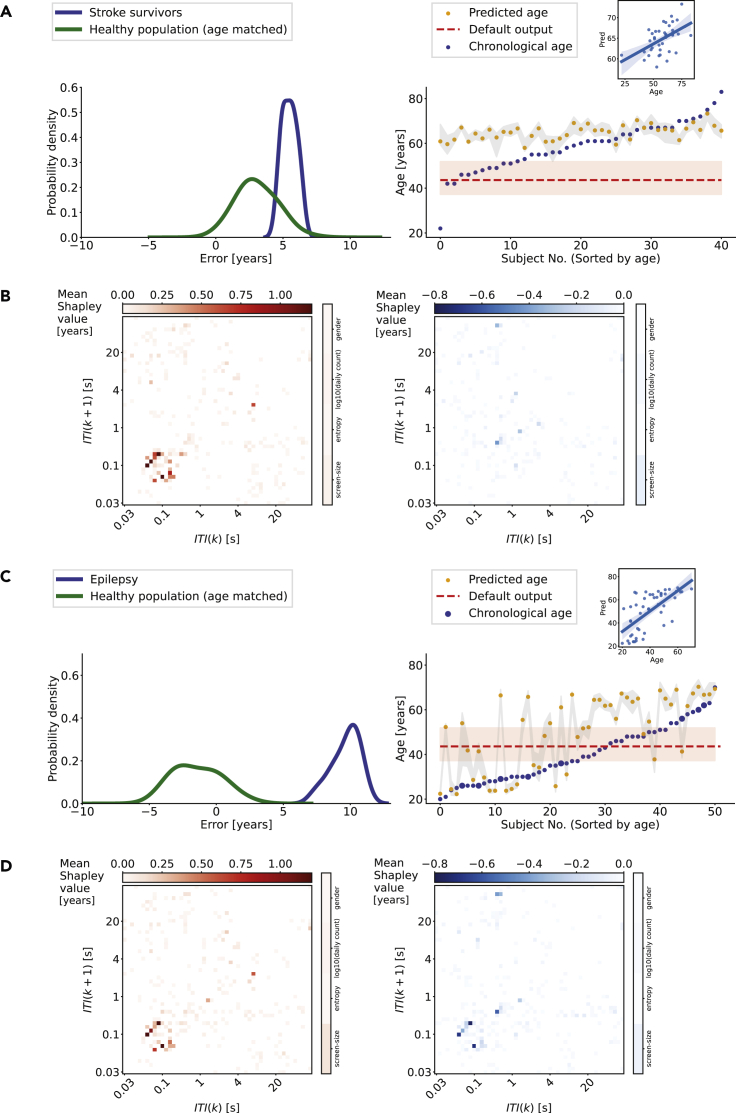
Deviations from healthy aging in stroke and epilepsy (A) We deployed the normative model based on healthy individuals on stroke survivors. The distribution of typical (median) errors based on healthy individuals age-matched to the stroke survivors (10,000 iterations, in green). The distribution of errors from the same model was obtained from stroke survivors (N = 41, 10-folds). Further examination of the model performance at the level of each stroke survivor reveals that accelerated aging was pronounced in survivors under 60 years of age. In stroke survivors, the model output was weakly correlated with the real age (insert). The shaded areas represent the 95% confidence intervals. (B) The (mean Shapley value across the population) importance of the different features in estimating the chronological age of stroke survivors, captured using the SHAP method, the contributions are separated in positive (red) and negative (blue). (C) The normative model is deployed in people with epilepsy. The distribution of errors was shifted indicating advanced age in epilepsy (N = 51) in contrast to the age-matched healthy population. Further examination at the level of each person with epilepsy indicated advanced aging across distinct ages. In people with epilepsy, the model output was moderately correlated with the real age. Persons implanted with the responsive neurostimulator (RNS) are marked using larger filled circles. The distribution of errors was smoothed with a Gaussian kernel (bandwidth 0.6) for display. The shaded areas represent the 95% confidence intervals. (D) The (mean Shapley values across the population) importance of the different features in estimating the chronological age of people with epilepsy, captured using the SHAP method, the contributions are separated in positive (red) and negative (blue).

We deployed the normative model in people with stroke who were able to operate smartphones. The majority of the survivors had a cortical stroke (35.7%), followed by a subcortical (26.2%), brain stem (28.6%), and cerebellar (3%) stroke. The time elapsed from the stroke was 28 days (median). The XGBoost model prediction error in the stroke survivors—who were between 22 and 83 years of age—was 5.47 years (mean across 10-folds, Figure 2). We first contrasted this error to the model predictions based on age-matched healthy people using an iterative subject selection (10,000 iterations). The ME across these iterations was 3.05 years, with the healthy and stroke distributions well-separated from each other (*t* = 5.21, p = 1.96 × 10^−7^, two samples *t*-test). Subsequently, we analyzed the predictions at the level of each patient by comparing the model-predicted age with the real age. Although the model performed poorly (MAE: 9.02 years), the predicted and real ages were weakly correlated with each other (*t* (39) = 3.62, p = 0.0008, R^2^ = 0.25, robust linear regression). Interestingly, the model estimated an age of ∼60 years for the stroke survivors, and this was distinct from the default model output based on null inputs (Figure 2).

The Shapley values helped determine which behavioral patterns contributed to the advanced age estimate in stroke (Figure 2). The two-dimensional bins capturing the fast (sub-second) consecutive intervals showed mostly positive Shapley values and marginal negative values, indicating that these patterns were altered in diseases contributing to the advanced aging predictions (see Figure S2 for single subject attributions).

### Age estimation using the normative model in people with epilepsy

We used the same approach as in the stroke survivors to estimate behavioral aging in people with epilepsy. The majority of the seizure foci involved the temporal lobe (41 of 51 patients); this includes onset zones spanning multiple lobes (e.g. frontotemporal). An extratemporal focus was present in 10 of the people with epilepsy (1 occipital, 3 parietal, 3 frontal, 2 insular, and 1 amygdala and thalamus). Eight of these subjects were implanted with a responsive neurostimulator and reported previously (Duckrow et al., 2021). The error of the model in people with epilepsy—who were between 20 and 70 years of age—was 9.64 years (mean across 10-folds, Figure 2). In age-matched healthy people (10,000 iterations) the ME was −1.43. The error distributions were well separated from each other (*t* = 20.26, p = 1.64 x 10^−89^, two samples *t*-test). Next, we focused on the predictions at the level of each patient by contrasting the predicted age with the real age. As in stroke, here too the model performed poorly (MAE: 12.79 years), but the predicted and real ages were moderately correlated with each other (*t* (49) = 5.99, p = 2.36 x 10^−7^, R^2^ = 0.42, robust linear regression). Unlike in stroke, where the model output typically approached ∼60 years, the model outputs in epilepsy covered the entire age range (Figure 2).

As for the patients with stroke, here too we used Shapley values to better understand which behavioral patterns underlay the advanced aging estimates. The two-dimensional bins of the JID capturing the short consecutive intervals mostly contained positive values, indicating that these patterns contributed to the advanced age predictions in people with epilepsy (see Figure S3 for single subject attributions).

## Discussion

A decision tree regression model performed well to infer the chronological age by using smartphone behavioral inputs in healthy individuals. The performance of the model was on par with age-estimating models based on biological inputs such as structural scans of the brain or blood samples. The transformation of smartphone behavior as a measure of healthy aging enabled us to address the nature of behavioral aging in two neurological diseases. In both epilepsy and stroke, the model estimated a more advanced age as if the diseases induced older behavior than the behavior in healthy aging.

In healthy individuals, the interpretable nature of the machine learning model could be used to estimate which aspects of the behavioral dynamics captured in the 2,500-bins large feature space contributed to the age estimation. The fast consecutive intervals were particularly useful to the model, albeit other behaviors covering a broad range of dynamics were also used. By using a range of cognitive tests, we recently found that the probability of fast consecutive intervals was correlated with sensorimotor, memory, and executive processes (Ceolini et al., 2022). The important role of the fast consecutive intervals in our model may stem from the convergence of multiple cognitive processes necessary to generate these behaviors.

Our finding that behavioral age in epilepsy and stroke is “older” than in healthy aging parallels the rich number of studies based on structural MRI of the brain that similarly demonstrated advanced aging in neurological diseases (Baecker et al., 2021). Interestingly, in stroke survivors and people with epilepsy, the MRI-based age was found to advance irrespective of the diverse brain regions at the disease epicenter (Cole et al., 2017; Sone et al., 2021). Similarly, in this study, diverse forms of stroke and epilepsy and corresponding medical histories were included, yet the aging appeared advanced irrespective of the detailed clinical descriptors. This pattern suggests that both brain and behavioral aging stem from more complex network changes and that the impact of the disease does not remain localized to specific neural or behavioral processes.

The extent of aging was advanced by ∼ 10 years in people with epilepsy in contrast to the advance of ∼6 years in stroke survivors (note, in the healthy group age-matched to the stroke group there was an advance of ∼3 years). A similar pattern of results also appears in the MRI-based age estimates such that the extent of advanced aging in people with epilepsy is more pronounced than that in stroke (Baecker et al., 2021). We speculate that the relatively smaller advanced aging in stroke is partly due to the conflation between the changes induced by natural aging and the disease.

Day-to-day behavior captured on the smartphone is a rich and largely unexploited source of information on aging in health and its perturbation in neurological disease. Limited smartphone behavioral data—spanning just 5 days—contained age-related information and suggest that our approach may even be applicable in settings where longer-term data acquisition is not possible. The parallels between age estimation using smartphone behavior and structural scans of the brain indicate that our behavioral approach is a widely accessible complement to brain measurements. Capturing behavioral patterns of healthy aging on the smartphone may help understand the behavioral alterations that occur in diseases.

### Limitations of the study

The decision tree regression model to capture healthy aging was trained on a Dutch sample spanning the adult life span. For this model to be used generally, broader sampling would be needed cutting across demographics and cultures. Furthermore, there were relatively sparse samples from middle-aged adults, and better sampling of this range is expected to improve the model performance. The self-reported healthy cohort could be further characterized according to lifestyle assessments and genetic susceptibilities to capture the spectrum of health instead of using a simple binary label of healthy versus unhealthy. The model may show a considerable loss of performance over the years because of the ever-shifting behavioral trends such as the use of a swiping keyboard, and it may be necessary to routinely update the model with new data samples. Toward the modeling, the smartphone behavioral features were captured using a JID based on the next-interval dynamics. Alternatives to the JID may be further explored to help improve the model performance. A few possible alternatives are to consider one-after-the-next-interval dynamics and include the type of app in use. Finally, a larger sample of people with stroke and epilepsy along with a more detailed analysis of the clinical status (as opposed to merely using the diagnosis labels as conducted here) may help better understand why some individuals showed more advanced behavioral age than others.

## STAR★Methods

### Key resources table

**Table:** 

REAGENT or RESOURCE	SOURCE	IDENTIFIER
**Software and algorithms**		

Python version 3.8	Python Software Foundation	https://www.python.org
MATLAB	MathWorks	https://www.mathworks.com/
KernelDensity, AdaBoost from sklearn v0.24.1	Pedregosa et al., (2011)	https://scikit-learn.org/stable/
XGBoost (Gradient Boosting)	Distributed (Deep) Machine Learning Community Chen and Guestrin, 2016	https://github.com/dmlc/xgboost
SHAP (SHapley Additive exPlanations)	Lundberg and Lee (2017)	https://github.com/slundberg/shap
Data processing, model definition and statistical analysis	This paper	https://github.com/codelableidenvelux/BehavioralAge_2022

### Resource availability

#### Lead contact

Further information and requests for code and data should be directed to and will be fulfilled by the lead contact Arko Ghosh (a.ghosh@fsw.leidenuniv.nl).

#### Materials availability

This study did not generate any materials.

### Experimental model and subject details

#### Participants

Self-declared healthy participants were recruited via the ongoing agestudy.nl data collection platform aided by a Dutch repository of volunteers for research (Ceolini et al., 2022; Zwan et al., 2021). The recruitment site usage locations included The Netherlands and Denmark. Previously reported data gathered via e-mail and in-person recruitments conducted primarily on campus in The Netherlands were additionally included (Borger et al., 2019; Huber and Ghosh, 2021; Westbrook et al., 2021). All participants provided informed consent and the data was gathered according to protocols approved by the Ethical Committee at the Institute of Psychology at Leiden University. The inclusion criteria were: ≥ 16 years of age, owned an (unshared) smartphone running the Android operating system, no neurological or mental health disorder (self-reported), no permanent injuries of the fingers, and access to a computer (public or private). Some of the participants opted to address the quality of life questionnaire (SF-36), from which mental and physical summary scores were derived (Laucis et al., 2015).

Participants with stroke were recruited as part of the ongoing QuantStroke study, (General Data Protection Regulation Aarhus University, Denmark, reference number 2016-051-000001, serial number 1766). Participants were offered participation while they were admitted to acute treatment or rehabilitation for first-ever or recurrent stroke. The included patients suffered from different levels of motor and cognitive impairments. They had to be ≥ 18 years with expected discharge to home and the ability to comply with study procedures including operating a smartphone. All participants provided informed written consent.

Participants with epilepsy were recruited from Stichting Epilepsie Instellingen Nederland (SEIN) and Kempenhaeghe in the Netherlands as part of the ongoing study ‘*Using day-to-day behavior on smartphones to improve epilepsy management’* (NCT04617418). This study was approved by the medical ethical committee Leiden-the Hague-Delft (METC LDD). Patients were screened by their treating neurologist at the outpatient clinics and eligible for inclusion in the study if they were 18 years old or over, diagnosed with refractory focal epilepsy and the diagnosis was supported by EEG and/or MRI abnormalities, had a high seizure frequency (≥1 per month), had one seizure type or multiple seizure types corresponding to one onset zone, had daytime seizures, were mentally competent and able to keep a seizure diary. All participants provided informed written consent. Eight epilepsy patients implanted with the responsive neurostimulation (RNS, Neuropace, Mountain View) were additionally included and the details of these patients have been reported previously (Duckrow et al., 2021).

#### Participant selection

As the data collections were part of ongoing studies, for instance, 3 years for the agestudy.nl platform. The number of participants used here is according to the database frozen on 27^th^ October 2021.

Towards the healthy data pool, we considered a total of 804 participants of which 636 were recruited via the agestudy.nl platform. Of the 804 recruits, 719 successfully installed the smartphone data logging app and reported their age in terms of the month and year of birth (259 males, 449 females, 11 unreported or unclear gender reports, 16 to 86 years of age). From this cohort, we analyzed 684 (median age 52 years, median recording duration 173 days) participants based on a cut-off of 7 days of smartphone recordings. Of this subset, 351 completed the quality of life SF-36 questionnaire.

Forty-one stroke survivors were successfully recruited based on a cut-off of 7 days of smartphone recordings. The population was aged between 22 and 83 (26 males, median age 60 years, median recording duration of 90 days). Fifty-two people with epilepsy were included based on a cut-off of 7 days of smartphone recordings. The population was aged between 20 and 70 (25 males, median age 38 years, median recording duration 160 days.

### Method details

#### Smartphone behavioral recordings

Day-to-day smartphone behavior was recorded using a background app (on Android operating smartphones), TapCounter (QuantActions, Lausanne)(Balerna and Ghosh, 2018). The app recorded the timestamps of all touchscreen interactions with ∼ millisecond resolution. The data was routinely transferred from the device to a cloud-based server and downloaded via the taps.ai (QuantActions, Lausanne) data collection manager. The data were next parsed using custom-written scripts using Matlab (MathWorks, Natick). The parsed data was used to estimate the joint-interval distribution of interactions (Duckrow et al., 2021). In brief, we first accumulated inter-touch intervals (ITI) over a period. We then created a 2D space by relating each ITI (at time *k*) against its subsequent interval (at time *k* + 1). We then operated 2D kernel density estimation (Gaussian kernel with a bandwidth of 0.1) over the log_10_-transformed 2D space generated by ITIs at time *k* and their subsequent ITIs at time *k* + 1. This step allows us to estimate the joint probability distribution of two subsequent ITIs (thus of three subsequent smartphone interactions). We then discretised the output of the kernel density estimation using 50 bins per dimension from 10^0.5^ ms to 10^5^ ms thus obtaining a 50 x 50 feature matrix for each individual.

#### The machine learning model of healthy aging

To estimate chronological age (dependent variable), we used the following independent variables: the self-reported gender (0 males, 1 female), the 2500 bins of the smartphone behavioral JID, the corresponding entropy, the number of smartphone interactions (log_10_ of the median across days) and the screen size (derived from the manufacturer’s record based on the smartphone model name).

We used XGBoost (Extreme Gradient Boosting) – a scalable machine learning system – for gradient tree boosting (Chen and Guestrin, 2016; Friedman, 2001; Pedregosa et al., 2011). The general objective of XGBoost used for regression problems is to build a tree ensemble model based on *K* additive functions that are weak decision trees. The algorithm works as follows, a first decision tree is fitted, subsequent decision trees are fitted by following second order steepest gradient descent on a differentiable cost function (e.g., mean squared error) and are trained to fit the residuals (prediction errors) obtained after applying the previously fitted trees. A learning rate is used to scale the residuals to fit, in particular, lower learning rates help reduce overfitting. After a fitting step, the predictions are scaled o their original range to obtain a final prediction which is improved w.r.t the previous step. The algorithm continues to improve the final prediction by adding the prediction of newly fitted trees until it reaches a pre-determined number of trees. The ideal number of trees is reached when more trees do not improve significantly the result and before the model starts overfitting. XGBoost uses several regularization techniques such as L1-norm, L2-norm and column-subsampling (Breiman, 2001). Moreover, XGBoost uses pruning of trees by clustering residuals into leaves (when determining branch splits) based on a scoring mechanism similar to impurity-based scoring. If the score gain in the group of leaves does not exceed a pre-defined minimum gain w. r. t. the parent leaves, the group is pruned. This helps avoid overfitting. Using 10-fold cross-validation and grid search we determined the following best parameters. A maximum tree depth of 9, 567 trees, the learning rate of 1*e*^-2^, minimum child weight of 8, L1-norm alpha parameter of 1*e*^-3^, column sub-sampling of 1.0, and row sub-sampling of 1.0. All the other parameters are kept at the default value (for detailed values see https://xgboost.readthedocs.io/en/stable/parameter.html).

Feature importance was estimated using Shapley values (derived from SHAP), which is based on a game theoretic approach to explaining the output of any machine learning model. It combines the methods of credit allocation and local explanations (Lundberg and Lee, 2017; Lundberg et al., 2020). For regression problems, given an input and a trained model, SHAP produced an explanation that consists of a positive or negative value for each feature of the input representing the contribution of that feature to the output.

### Quantification and statistical analysis

#### Contrasting model performance in health vs. neurological diseases

We trained 2 batches of 10 models with 10-fold cross-validation using the data from the healthy population. The first batch was trained with input data coming from the healthy population and corresponding to JIDs obtained from smartphone usage accumulated over a maximum of 90 days. This batch was used for the comparison between the healthy population and the stroke survivors' population, the maximum of 90 days was chosen to match the monitoring duration period of stroke survivors. The second batch was trained with input data coming from the healthy population and corresponding to JIDs obtained from smartphone usage accumulated over a maximum of 180 days. This second batch was used for the comparison between the healthy population and the epilepsy population.

We used bootstraps to compare the distribution of errors across age-matched populations of diseased vs. healthy populations. Each batch had a total of 10 trained models, obtained from the 10-fold cross-validation operation. With the first batch, we obtained ten different age predictions for people with stroke or epilepsy. We estimated the typical (median) error made by each model resulting in 10 values per batch. To contrast these errors to the healthy population, we iteratively selected the same number of participants from the healthy population such that they were age-matched to the stroke or epilepsy population. The median error was gathered for each of the 10,000 iterations. The distributions were compared using two-sample *t*-tests.

## Data Availability

• The analyzed data from the level of the JID and the associated age, public id to connect with other studies using the same smartphone data, and gender information is made available on dataverse.nl within a month after the publication according to the Leiden University Institute of Psychology guidelines. • The codes used to analyze the links between JID and age are deposited on the Leiden University Cognition in the Digital Environment (CODELAB) git repository https://github.com/codelableidenvelux/BehavioralAge_2022. • Any additional information required to reanalyze the data reported in this paper and shared is available from the lead contact upon request.
