# Supplementary material for: A model of healthy aging based on smartphone interactions reveals advanced behavioral age in neurological disease
Source: iScience. 2022 Aug 5;25(8):104792. doi: 10.1016/j.isci.2022.104792 (PMC9418593; doi:10.1016/j.isci.2022.104792)
Supplement: Document S1. Figures S1–S3 [file mmc1.pdf]

iScience, Volume *25*

Supplemental information

A model of healthy aging based on smartphone interactions reveals advanced behavioral age in neurological disease

Enea Ceolini, Iris Brunner, Johanna Bunschoten, Marian H.J.M. Majoie, Roland D. Thijs, and Arko Ghosh

A model of healthy aging based on smartphone interactions reveals advanced behavioral age in neurological disease

July 11, 2022

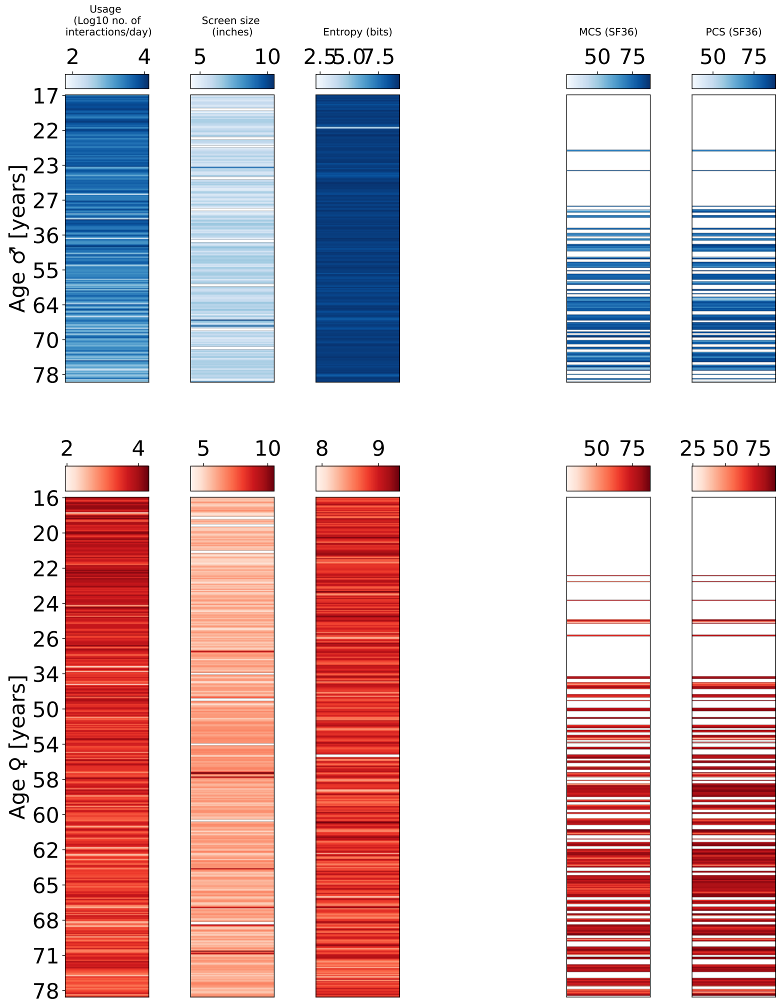

Figure S1: *Smartphone-related measures considered here apart from the JID, and the SF-36 outcomes*: The data is sorted by gender and age. White spaces indicate unavailable data. The first three columns were derived using objective measures, whereas the later two columns are based on self-reports, related to Figure 1.

.

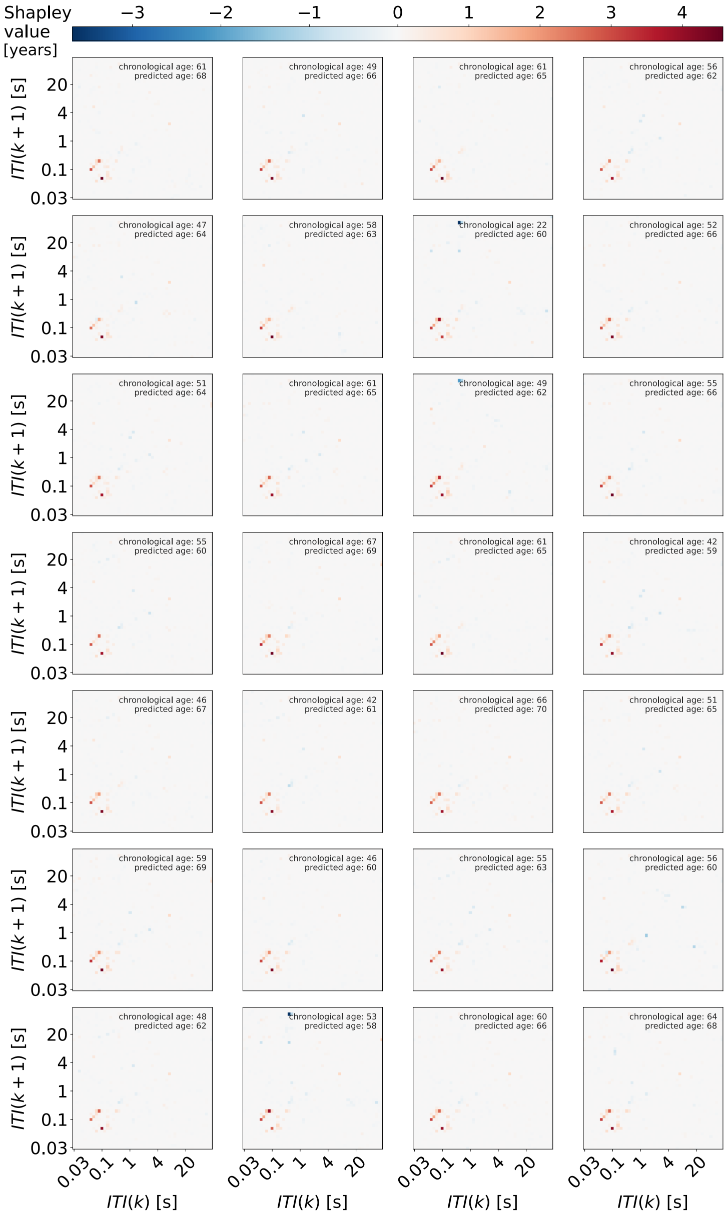

Figure S2: *Subject-specific Shapley attributions for stroke survivors.* Shapley attributions (estimated using the SHAP toolbox) for those stroke survivors that show accelerated aging, explaining the contribution of each input feature to the final predicted age, related to Figure 2.

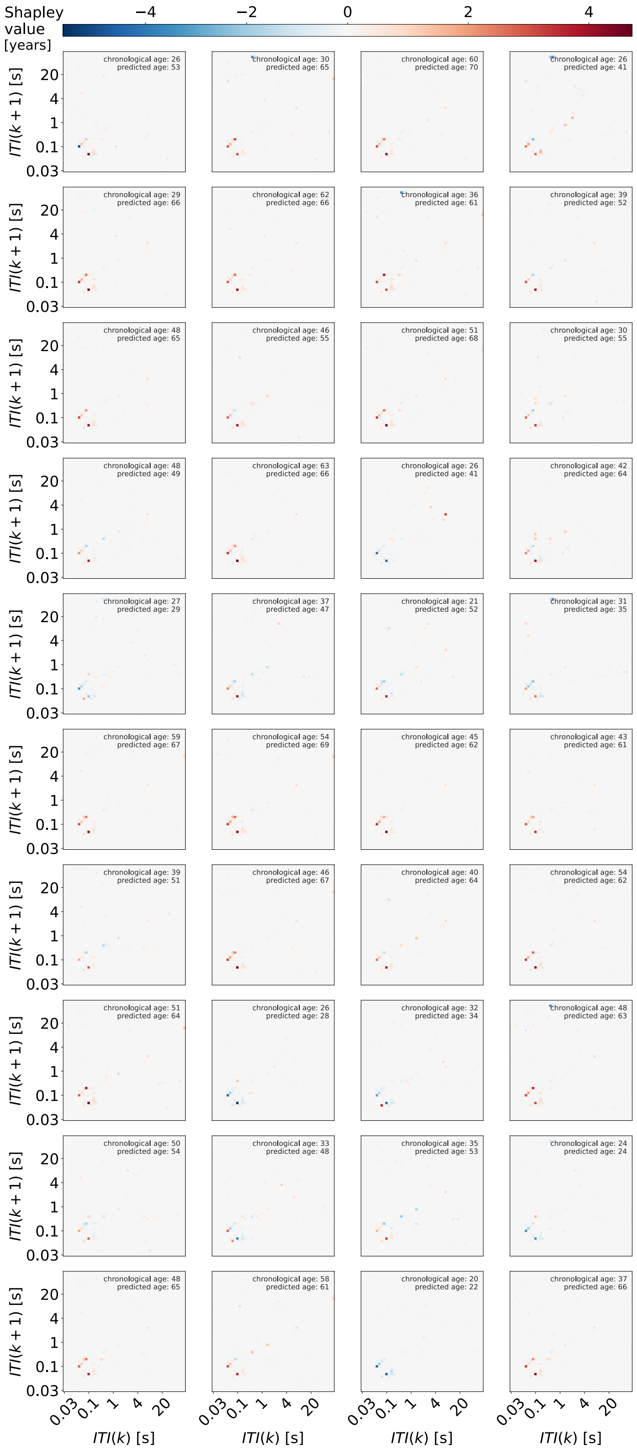

Figure S3: *Subject-specific Shapley attributions for people with epilepsy.* Shapley attributions (estimated using the SHAP toolbox) for those epilepsy subjects that show accelerated aging, explaining the contribution of each input feature to the final predicted age, related to Figure 2.
